# Effectiveness of isometric muscle training combined with manual lymphatic drainage on secondary lower extremity lymphedema following gynecologic cancer surgery

**DOI:** 10.3389/fbioe.2025.1568003

**Published:** 2025-05-14

**Authors:** Jiahui Ma, Hewei Wang, Yilan Li, Xiang Guo, Mengjia Xie, Xinxin Wang, Luxi Mao, Dapeng Xing, Li Shen, Dan Chen, Jingxin Wang

**Affiliations:** ^1^ Department of Rehabilitation Medicine, Zhengzhou Central Hospital Affiliated Zhengzhou University, Zhengzhou, China; ^2^ Department of Rehabilitation, Huashan Hospital, Fudan University, Shanghai, China; ^3^ Department of Rehabilitation, Hangzhou Linping Hospital of Traditional Chinese and Western Medicine, Hangzhou, China; ^4^ Department of Rehabilitation Medicine, Jing’an District Central Hospital of Shanghai, Shanghai, China

**Keywords:** secondary lymphedema, gynecologic cancer, lower extremity, isokinetic strength training, manual lymphatic drainage

## Abstract

**Objective:**

To investigate the effects of isokinetic strength training combined with manual lymphatic drainage (MLD) on leg circumference, walking ability and muscle strength in patients with secondary lymphedema following gynecologic cancer surgery.

**Design:**

Randomized controlled trial.

**Setting:**

Inpatient rehabilitation department.

**Participants:**

Sixty-six patients with secondary lymphedema of the lower extremities following gynecologic cancer surgery were randomly allocated into an experimental group and a control group, each comprising 33 patients.

**Interventions:**

The control group participated in a 4-week standardized MLD program. In addition to the MLD program, participants in the experimental group received additional isokinetic strength training for 20 min daily over the same 4-week period.

**Outcomes:**

Lower limb volume derived from the circumference measurements, Holden Gait Scale and Lovett muscle strength grading.

**Results:**

Prior to the intervention, no statistically significant differences were observed between the two groups across all outcomes (P > 0.05). Post-intervention, statistically significant improvements were noted in the experimental group compared to the control group with respect to reduced lower extremity volume, improved walking ability, and increased muscle strength (P < 0.05).

**Conclusion:**

For patients with secondary lower limb lymphedema following gynecological tumor surgery, a combination of isokinetic strength training and MLD has been found to be more effective than MLD alone in reducing edema, improving walking ability, and enhancing muscle strength.

## Introduction

A common complication of gynecological cancer treatment is secondary lower limb lymphedema, mainly affecting patients with cervical, endometrial, and ovarian cancers after surgery, radiotherapy, or chemotherapy (Bitler and Carpenter, 2017; Ida et al., 2019). Postoperative disruption of pelvic lymphatic drainage may result in the accumulation of lymphatic fluid in the lower extremities, ultimately leading to lymphedema (Caretto et al., 2022). According to a report by the Chinese Academy of Sciences, the incidence of secondary lower limb lymphedema as a complication following cervical cancer surgery ranges from 11.5% to 30.6% (Liu et al., 2023). Lower limb lymphedema can cause symptoms like heaviness, pain, muscle weakness, abnormal sensations and anxiety, significantly impacting the patient’s quality of life (Clinckaert et al., 2022) and causing substantial burdens and mental stress for both patients and their families (Ciudad et al., 2019; Dean et al., 2019).

For lower limb lymphedema, current treatments include conservative therapy and surgery (Reichart, 2017). Complex Decongestive Therapy (CDT) is a prominent non-surgical intervention for the management of lymphedema and is extensively utilized in clinical settings (Brandão et al., 2020). CDT consists of two phases: the intensive treatment phase and the maintenance phase. The intensive phase involves education, compression therapy, decongestive exercises, and manual lymphatic drainage (MLD). The maintenance phase focuses on sustaining and enhancing these results through compression garments, skin care, and home exercises (Liu et al., 2021). Although CDT has been extensively applied and studied for the management of upper limb lymphedema, its application for lower limb lymphedema remains underexplored, with insufficient evidence-based support. MLD, a critical component of CDT, entails the gentle facilitation of lymphatic fluid movement from congested regions to unaffected vessels through soft manual therapy (Thompson et al., 2021; Ali et al., 2022). The efficacy of MLD in the management of lymphedema has been demonstrated in observational studies and certain randomized controlled trials (RCTs). Nevertheless, there remains a paucity of high-quality RCTs with adequately powered sample sizes, especially for the management of lower limb lymphedema (Executive Committee of the International Society of Lymphology, 2020; Liang et al., 2020; Zhang et al., 2022).

Although limb lymphedema can be alleviated through MLD, a passive method to facilitate lymphatic drainage, active exercise is also crucial for increasing lymph fluid production and drainage, leading to more satisfactory long-term results (Wang and Dong, 2019). Research shows that isokinetic strength training, by activating muscles and enhancing contractions, increases pressure on lymphatic vessels. This promotes blood circulation and helps reduce edema by facilitating the absorption and drainage of interstitial fluid in patients (Zhu et al., 2023; Petrucci et al., 2024). Isokinetic strength training provides consistent muscle resistance throughout the range of motion, maintaining optimal muscle tension and ensuring thorough exercise (Wang et al., 2022). Strength training modulates sympathetic nerve activity, inducing self-contraction of lymphatic vessels. This mechanism is crucial for the long-term management of lymphedema (Omar et al., 2020). Recent systematic reviews have indicated that resistance strength training does not increase the risk of lymphedema or exacerbate its symptoms; instead, it improves physical function and quality of life (Baumann et al., 2018). These findings alleviate safety concerns regarding the application of isokinetic strength training for patients with lymphedema.

Given the limited reports on using isokinetic strength training with MLD for treating secondary lower limb lymphedema due to gynecological malignancies, we designed a randomized controlled study to evaluate the efficacy of this combined therapy. We hypothesized that combining isokinetic strength training with MLD would reduce lymphedema more effectively and improve lower limb mobility in these patients.

## Materials and methods

### Participants

Sixty-six patients with secondary lymphedema following gynecological malignant tumor surgery, admitted to the Rehabilitation Department of Zhengzhou Central Hospital from July 2022 to July 2023, were selected as research subjects. All participants provided written informed consent. This study was reviewed and approved by the Ethics Committee of Zhengzhou Central Hospital (approval number: 202,256). This study adhered to the principles of the Declaration of Helsinki and was conducted and reported in accordance with the recommendations provided by the CONSORT guideline (Schulz et al., 2010).

### Inclusion/exclusion criteria

The inclusion criteria were the following: (1) Females diagnosed with unilateral lower limb lymphedema according to The diagnosis and treatment of peripheral lymphedema: 2020 Consensus Document of the International Society of Lymphology (Executive Committee of the International Society of Lymphology, 2020); (2) with unilateral lower limb lymphedema based on ≥5.7% circumference difference between affected and contralateral limbs (Campanholi et al., 2015; Cormier et al., 2010); (3) a history of lymph node dissection; (4) no deep vein thrombosis; (5) ability to cooperate with functional exercises, provide informed consent, and participate voluntarily.

The exclusion criteria were: (1) lower limb edema from cardiac, renal, or malnutrition causes; (2) lower limb venous thrombosis or superior vena cava obstruction; (3) acute cellulitis, erysipelas, or severe lower limb infection; (4) unstable hypertension; (5) untreated tuberculosis or malaria; (6) cognitive or behavioral issues preventing cooperation with treatment.

### Randomization

Participants meeting the inclusion criteria were randomly allocated to either the experimental group (n = 33, MLD + isokinetic strength training) or the control group (n = 33, MLD alone). Baseline assessments were conducted post-randomization. A computer-generated random number list, managed by a researcher unaffiliated with participant recruitment or the study site, ensured allocation concealment. An independent investigator, not involved in randomization, intervention, or evaluation, recruited all participants. The allocation sequence remained concealed from all researchers and assessors.

### Prior sample size estimation

The sample size was determined based on previous studies. We assumed a mean difference of 400 cm^3^ between groups in the changes of lower limb volumetric discrepancy between the affected and unaffected limbs, with a common standard deviation of 500 cm^3^. Anticipating a 10% dropout rate, we calculated a final target sample size of 33 participants, allocating 33 to each group to achieve 85% power at a 0.05 significance level.

### Intervention

Participants underwent a 4-week inpatient intervention for lower extremity lymphedema. Both groups received the MLD program administered by therapists certified in international lymphedema therapy and comprehensive decongestive therapy (CDT). Additionally, the experimental group received isokinetic strength training in conjunction with MLD.

### MLD

Conventional MLD technique were employed, utilizing four basic techniques: stationary circle, rotary technique, pump technique and scoop technique. The treatment began at the proximal end of the limb, then moved to the distal areas, and finally returned to the proximal end, following this sequence (Table 1) (Földi and Strossenreuther, 2005).

**TABLE 1:** MLD procedure.

Lumbar massage: The patient lies in a prone position, with the therapist standing at the side	
①	Effleurage: Gently pressing with the hand
②	Trunk side: Applying stationary circular pressure on the side of the trunk towards the inguinal lymph nodes
③	“L-shaped” technique: Starting from the lumbar spinous process and moving laterally across the trunk in a rotary massage, then performing stationary circles towards the highest point of the pelvis, directing pressure towards the inguinal lymph nodes
④	Lateral gluteal area treatment: The therapist performs stationary circles along three main lines on the side of the buttocks, with multiple starting points, pressing towards the inguinal lymph nodes
⑤	Central gluteal area treatment: Massaging the central drainage area of the thigh, pressing towards the central inguinal lymph nodes
⑥	Paraspinal treatment: Performing stationary circles on both sides of the spine

Leg Massage: The patient should be in a supine position, with the therapist standing at the side	
①	Effleurage: Gently pressing in the direction of drainage
②	Inguinal lymph node treatment: Starting the massage of the inguinal lymph nodes from three different locations
③	Thigh treatment: Begin with stationary circles in the central anterior area of the thigh, then apply pumping pressure in the same area, and finally alternate between stationary circles and pumping pressure on the anterior and lateral sides of the thigh, always directing towards the inguinal lymph nodes
④	Knee treatment: Start with pumping pressure around the patella, then perform stationary circles on the popliteal lymph nodes while simultaneously massaging with the palm down, followed by stationary circles on the central knee area, and finally stationary circles below the fibular head
⑤	Calf treatment: With the patient’s knee bent, the therapist performs “scoop-like” pressure on the gastrocnemius with one hand while the other hand performs pumping pressure on the tibialis anterior, then use both hands for “scoop-like” pressure. If the leg cannot be bent, perform stationary circles instead
⑥	Foot massage: Begin with stationary circles below the ankle joint and along the Achilles tendon, then perform stationary circles on the dorsal side of the ankle joint while passively moving the ankle, followed by stationary circles on the dorsum of the foot, and finally stationary circles on the toes

During the MLD procedure, the following points should be noted: The drainage force should be gentle and consistent, with traction applied between the skin without friction, and the pressure should be minimal, just enough to avoid skin redness; the operation speed should be slow and rhythmic, with each phase (working and resting) lasting at least 2 s; each treatment session should last 40 min, once a day, five times a week, for a total of 4 weeks (Kasseroller, 1998).

### Isokinetic strength training

Isokinetic strength training was conducted using the LoopGO™ (China). Training sessions were performed on a bedside lower limb active-passive rehabilitation trainer, following an isokinetic prescription. Patients were placed in a supine position with hips and knees flexed, and their lower limbs were secured within the trainer’s groove. The movement speed and direction were adjusted to a moderate intensity based on individual patient tolerance and conditions. Each session lasted 20 min and was conducted twice daily, 5 days per week, over a 4-week period.

### Outcomes

All outcomes were assessed by an independent trained physician blinded to the grouping information, before and after 4 weeks of treatment. The primary outcome was lower limb circumference. Secondary outcomes included gait function and lower limb muscle strength.

## Lower limb volume derived from circumference measurements

**FIGURE 1:**
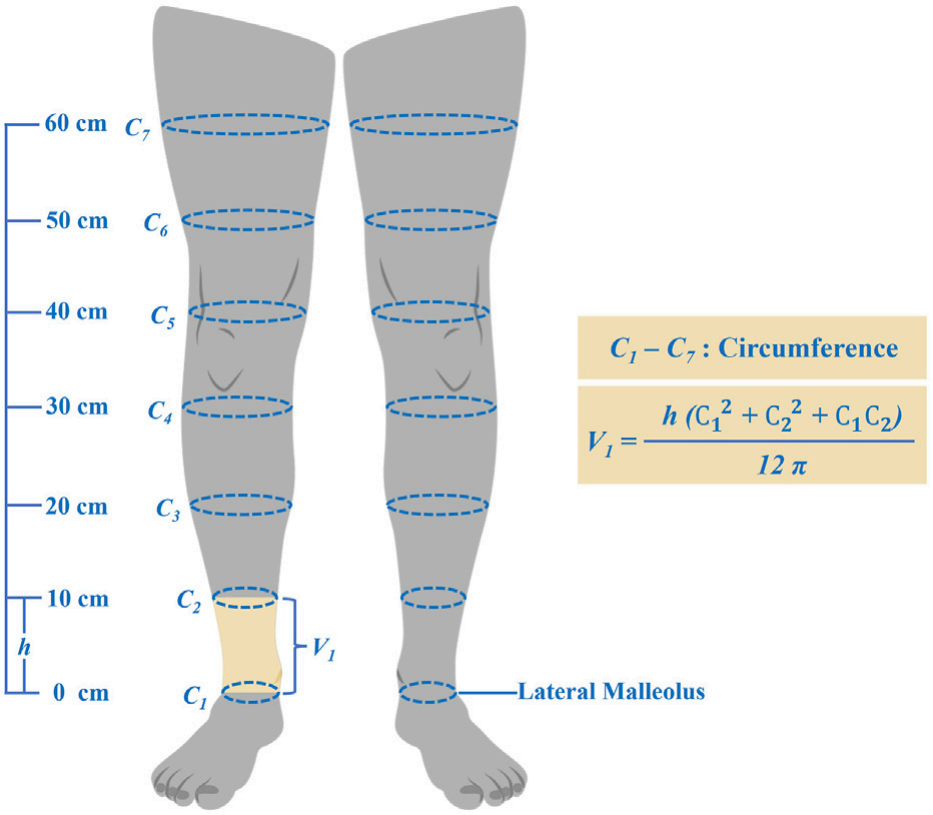
Lower limb circumferential measurement and volumetric calculation formula.

A tape measure was used to assess the circumference of the affected lower limb in both groups before and after treatment. Measurements were conducted by the same therapist to ensure consistency and accuracy. Patients were positioned supine on a treatment bed in neutral alignment. Using the lateral malleolus as a bony landmark, measurements were taken from the ankle joint proximally at intervals of 10 cm, 20 cm, 30 cm, 40 cm, 50 cm, and 60 cm. Each measurement was performed three times, with the median value being recorded. Lower extremity volume was calculated using a formula reported by Casley-Smith: V = h (C_1_^2^ + C_1_C_2_+ C_2_^2^)/12π, where V is the volume of an extremity segment, C_1_ and C_2_ are circumferences at each end, and h is the distance between the ends (Figure 1). The volumetric discrepancy between the affected and unaffected limbs was calculated both before and after treatment.

### Gait function assessment

The Holden Gait Scale, also referred to as the Functional Ambulation Categories (FAC), is a tool used to assess walking ability. This 6-level scale evaluates ambulation by determining the amount of human support required during walking, irrespective of assistive device use (Holden et al., 1984). Level 0 indicates non-functional ambulation (inability to walk). Levels I to III denote dependent ambulation, requiring assistance from another person: continuous manual contact (Level I), intermittent manual contact (Level II), or verbal supervision/guidance (Level III). Levels IV and V describe independent ambulation: walking on level surfaces only (Level IV) or on any surface (Level V).

### Muscle Strength Test

The Lovett muscle strength grading system was utilized for manual muscle testing (MMT) (Cuthbert and Goodheart, 2007). Patients removed clothing that could affect assessment outcomes. The therapist stabilized the patient’s trunk or limb in an optimal position to facilitate specific actions and prevent unintended joint movements, focusing on the calf muscle group (ankle plantar flexors). Depending on individual conditions, gravity, muscle contraction, resistance, and range of motion tests were applied. Muscle strength was graded on a 0–5 scale before and after treatment, with higher scores indicating greater strength. To ensure data accuracy, all pre- and post-treatment assessments were conducted by the same therapist.

### Statistical analysis

The statistical analysis was conducted by an independent statistician who was blinded to the interventions. The data were analyzed using SPSS (Version 26.0, Inc., Chicago, IL) statistical software. Continuous data were presented as the mean ± SD, while categorical variables were expressed as numbers (%). The homogeneity at baseline was verified through the implementation of the Student’s t-test, Wilcoxon rank-sum test and chi-square test. The normality of data distribution was assessed using the Shapiro-Wilk statistic. Independent samples t-tests were employed to evaluate differences between groups, while Wilcoxon rank-sum test was utilized in cases where values did not adhere to a normal distribution or for ordinal data. The significance level was set at α = 0.05. A per-protocol analysis was conducted, exclusively considering participants who successfully completed all assessments (Schoenfeld, 2005).

## Results

### Recruitment and retention

**FIGURE 2:**
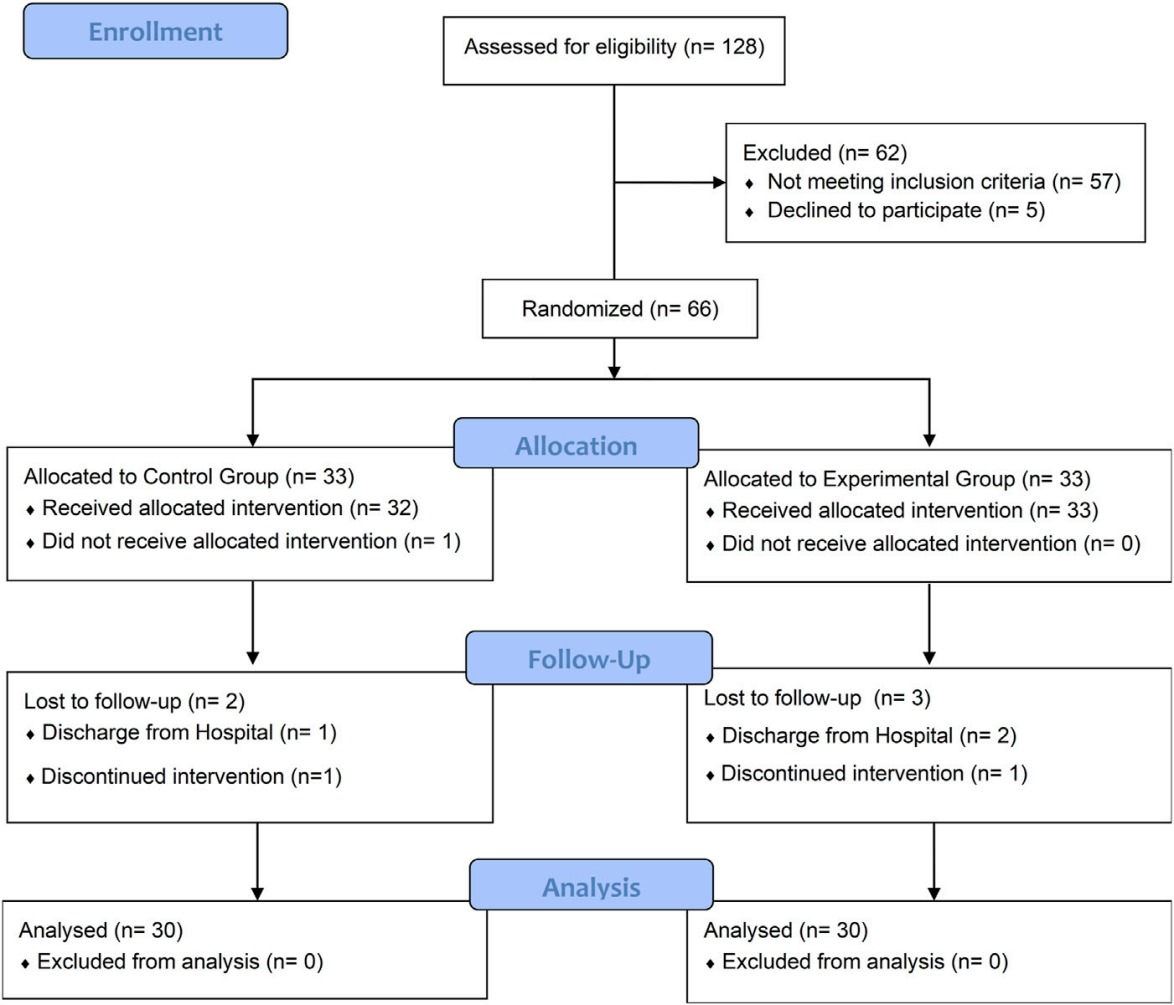
CONSORT flow diagram.

Among the initial pool of 188 patients who underwent eligibility screening, a total of 71 individuals met our study criteria, with 66 of them consenting to participate in this study and random equally allocated to the experiment and control group. At baseline assessment no significant differences (P > 0.05) were observed between the two groups in terms of baseline demographic characteristics and all outcomes (Table 2). The flow diagram (Figure 2) presents the detailed information regarding participant recruitment, allocation, and follow-up.

**TABLE 2:** Comparison of the demographic data of the two group.

Variables	Experimental group (n = 30)	Control group (n = 30)	t/Z/χ^2^	P
Age (year)	55.1 ± 14.41	53.13 ± 14.41	−0.626	0.534
Weight (kg)	66.65 ± 8.14	63.43 ± 8.73	−1.476	0.145
BMI	25.68 ± 3.61	23.93 ± 3.24	−1.978	0.053
Height (cm)	160 (157, 164)	161 (160,166.3)	−1.073	0.283
Time Since Edema (days)	31 (16.5, 61.8)	26.5 (13.3,35.3)	−1.45	0.147
Affected Side (L/R)				
Left Side	12 (40%)	12 (40%)	0.000	1.000
Right Side	18 (60%)	18 (60%)		

### Lower limb volume

Before treatment, there was no statistically significant difference the volumetric discrepancy between the affected and unaffected limbs between the two groups (P > 0.05). After treatment, the volumetric discrepancy between the affected and unaffected limbs in both groups decreased compared to before treatment, and the experimental group showed a greater decrease than the control group, with a statistically significant difference (P < 0.05), as detailed in Table 3.

**TABLE 3:** Comparison of the pre- and post-intervention volumetric discrepancy between the affected and unaffected limbs between the two groups( $\bar{x}\pm$ s, cm^3^ **)**.

Groups	n	Pre-intervention	Post-intervention	Pre-post
Experimental Group	30	1872.21 ± 1150.45	1010.00 ± 605.43	862.21 ± 757.99
Control group	30	1720.84 ± 1231.61	1215.50 ± 1173.74	505.34 ± 545.30
T		0.492	−0.852	2.093
P		0.625	0.398	0.041

### Gait function level score

**FIGURE 3:**
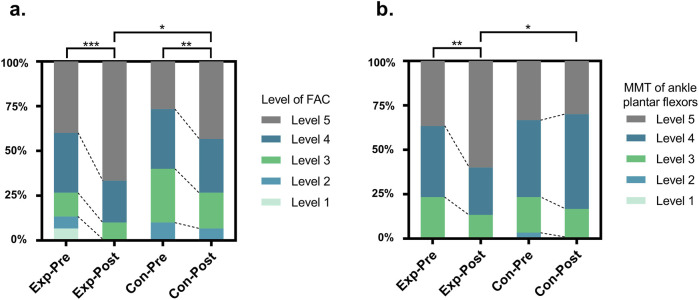
Changes of Gait Function Level Score **(a)** and Muscle Strength Test **(b)** between the groups before and after intervention. Exp, experimental group; Con, control group; FAC, Functional Ambulation Categories; MMT, Muscle Strength Test; *, P < 0.05; **, P < 0.01; ***, P < 0.001.

Before treatment, no statistically significant difference was observed in gait function level scores between the two groups (P > 0.05). After treatment, gait function level scores increased significantly in both groups compared to pre-treatment levels (experimental group, P < 0.001; control group, P = 0.002). Moreover, the experimental group exhibited higher scores than the control group, with a statistically significant difference (P < 0.05), as shown in Table 4 and Figure 3a.

**TABLE 4:** Comparison of walking ability (FAC) between the two groups before and after 4 weeks of treatment (%).

	Groups	n	Level 1	Level 2	Level 3	Level 4	Level 5
Pre-intervention	Experimental group	30	2 (6.7)	2 (6.7)	4 (13.3)	10 (33.3)	12 (40.0)
	Control group	30	0 (0.0)	3 (10.0)	9 (30.0)	10 (33.3)	8 (26.7)
	Z		1.004				
	P		0.315				
Post- intervention	Experimental group	30	0 (0.0)	0 (0.0)	3 (10.0)	7 (23.3)	20 (66.7)
	Control group	30	0 (0.0)	2 (6.7)	6 (20.0)	9 (30.0)	13 (43.3)
	Z		2.010				
	P		0.044				

### Muscle Strength Test

Before treatment, there was no statistically significant difference in the MMT of ankle plantar flexors on the affected side between the two groups (P > 0.05). After treatment, muscle strength increased in both groups compared to pre-treatment levels; however, a significant improvement was observed only in the experimental group (experimental group, P = 0.002; control group, P = 0.317). Furthermore, the experimental group exhibited higher scores than the control group, with a statistically significant difference (P < 0.05), as shown in Table 5 and Figure 3b.

**TABLE 5:** Comparison of strength of calf muscle groups (MMT, ankle plantar flexors) between the two groups before and after 4 weeks of treatment (%).

	Groups	n	Level 1	Level 2	Level 3	Level 4	Level 5
Pre-intervention	Experimental group	30	0 (0.0)	0 (0.0)	7 (23.3)	12 (40.0)	11 (36.7)
	Control group	30	0 (0.0)	1 (3.3)	6 (20.0)	13 (43.3)	10 (33.3)
	Z			0.237			
	P			0.813			
Post- intervention	Experimental group	30	0 (0.0)	0 (0.0)	4 (13.3)	8 (26.7)	18 (60.0)
	Control group	30	0 (0.0)	0 (0.0)	5 (16.7)	16 (53.3)	9 (30.0)
	Z			1.982			
	P			0.047			

## Discussion

Secondary lymphedema is a chronic, irreversible condition with rising incidence. Up to 47% of patients undergoing surgery for gynecological cancers develop lower limb lymphedema (Wong et al., 2022). Lymphatic vessel damage or disruption of lymphatic circulation caused by surgical procedures is difficult to repair spontaneously (Aoishi et al., 2020). Lower limb lymphedema significantly restricts physical activity, such as daily walking, standing, and performing household chores (de Sire et al., 2022; Tedeschi, 2023). Without timely intervention, such damage is highly likely to progress to irreversible soft tissue fibrosis (Nakamura et al., 2017). To date, research on lymphedema has predominantly focused on upper limb lymphedema in breast cancer patients. Given the notable differences in tissue composition and function between the upper and lower limbs, it is crucial to develop effective treatment strategies specifically for lower limb lymphedema (Biglia et al., 2017). In this study, we demonstrated that a 4-week regimen integrating MLD with isokinetic strength training led to a reduction in lower extremity volume, an enhancement in walking ability, and an increase in muscle strength compared to MLD treatment alone. Our results highlight the importance of incorporating strength training into the management of lower limb lymphedema, as it not only improves walking ability but also facilitates lymphatic absorption and drainage.

MLD is a non-invasive, safe, effective, and convenient therapeutic approach that has been widely adopted for managing swelling of various etiologies (Lin et al., 2022). MLD addresses secondary lymphedema in the lower limbs by employing techniques analogous to those utilized for the upper limbs. Through gentle massage and manual maneuvers, MLD facilitates the circulation of lymphatic fluid, thus alleviating edema symptoms caused by lymphatic system dysfunction (Thompson et al., 2021). Unlike compression bandages or garments, which frequently induce localized heat retention and perspiration (particularly in lower limbs), limit joint mobility during daily activities, MLD overcomes these limitations through non-occlusive, anatomically adaptive techniques that synchronize with natural lymphatic rhythms, thereby sustaining therapeutic efficacy without compromising patient comfort or functional independence (Liang et al., 2020). However, MLD as a standalone treatment has inherent limitations. Its reliance on operator expertise can lead to variability in treatment quality, while prolonged therapy poses logistical and financial challenges for patients. Furthermore, individual responses to MLD vary significantly due to factors such as disease chronicity and the severity of tissue fibrosis (Thompson et al., 2021; Ali et al., 2022). Notably, MLD passively enhances lymphatic transport and drainage; however, if patients actively improve lymphatic return through exercise, it may yield more effective outcomes, particularly for lower limb lymphedema.

Isokinetic strength training, a method specifically designed to improve muscle strength and endurance, has recently been applied to edema treatment and has demonstrated promising results (Petrucci et al., 2024). This training method enhances lymphatic flow through sustained muscle contraction and relaxation in an isokinetic manner, thereby promoting the drainage of lymphatic fluid and alleviating edema symptoms. Systematic reviews have highlighted that, despite the limited number and quality of studies currently available, exercise is not only feasible and safe but also exhibits clinically significant efficacy in alleviating lymphedema-related symptoms in women post-gynecological cancer surgery (Hsu et al., 2024; Wittenkamp et al., 2025). Zhang et al. (2021) demonstrated that progressive resistance exercise is feasible and safe for post-cervical cancer surgery lymphedema, with no serious adverse reactions and no equipment or regional limitations. Research has also demonstrated that combining isokinetic strength training with MLD offers notable advantages in managing upper limb lymphedema following breast cancer surgery (Wang and Dong, 2019). Additionally, research has demonstrated that progressive resistance exercise training serves as an effective approach to prevent lower limb lymphedema following pelvic lymphadenectomy for cervical cancer (Zhang et al., 2024). Through isokinetic strength training, the controlled contraction and relaxation of the affected muscles, along with the compression of deep tissues, contribute to enhanced blood circulation and lymphatic return. This process may also facilitate the clearance of algogenic factors, thereby alleviating lymphedema and associated discomfort in patients (Wittenkamp et al., 2025). Furthermore, isokinetic strength training has been shown to enhance muscle strength and endurance, thereby improving exercise capacity and daily activity levels in patients (Xu and Liu, 2024).

However, strength training exhibits certain limitations when employed as a sole intervention in the treatment of lymphedema. This is due to the multifactorial nature of lymphedema, which involves complex pathological mechanisms. Repeated strength-training sessions may become impractical for patients with moderate to severe lower limb lymphedema and could potentially exacerbate symptoms. This exacerbation may result from increased local tissue pressure caused by sustained muscle contractions (Hayes et al., 2009). Therefore, integrating isokinetic strength training with other therapies based on individual condition is essential for comprehensive management and rehabilitation. Evidence suggests that MLD can significantly alleviate discomfort associated with muscle training, such as delayed onset muscle soreness and increased local tissue pressure resulting from prolonged muscle contractions, which may exacerbate edema symptoms (Földi and Strossenreuther, 2005). Meanwhile, rhythmic skeletal muscle contractions during active exercise generate a pump-like mechanism, exerting intermittent pressure on lymphatic vessels and veins to enhance lymph drainage and venous return, thereby improving fluid transport efficiency in the lymphatic system (Hsu et al., 2024; Wittenkamp et al., 2025).

Our study also explored the effects of combining isokinetic strength training with MLD to improve lower limb function in patients with secondary lymphedema. Results showed that the experimental group also had better outcomes in Holden gait scores and Lovett scores compared to the control group. Isokinetic training, by adjusting resistance to match patient strength, maximizes muscle loading while preventing atrophy and scar contracture associated with gynecologic cancer surgery (Hanson et al., 2016). This treatment further enhances the resilience of connective tissues and muscle elasticity, thereby promoting patient mobility, which is crucial for individuals with secondary lymphedema following gynecologic cancer surgery (Figueira et al., 2023).

Our study also has certain limitations that warrant acknowledgment. For instance, the relatively small sample size and single-center design may limit the generalizability of our findings. Additionally, long-term follow-up data are lacking. Furthermore, this study did not include the use of bandages, which constitute a critical component of CDT. Future research should aim to address these limitations by conducting larger, multicenter trials with extended follow-up periods and by exploring the effects of MLD in combination with isokinetic strength training and bandage application.

## Conclusion

Following gynecological tumor surgery, patients who develop secondary lower limb lymphedema have shown greater benefits from a regimen that combines isokinetic strength training with MLD. This integrated approach has been more effective in reducing swelling, enhancing ambulatory function, and increasing muscle strength compared to MLD alone. Further research is essential to investigate its long-term efficacy and potential clinical applications.

## Data Availability

The original contributions presented in the study are included in the article/supplementary material, further inquiries can be directed to the corresponding author.
